# Can Chat-GPT read and understand guidelines? An example using the S2k guideline intrauterine growth restriction of the German Society for Gynecology and Obstetrics

**DOI:** 10.1007/s00404-024-07667-z

**Published:** 2024-08-05

**Authors:** Iason Psilopatis, Simon Bader, Annika Krueckel, Sven Kehl, Matthias W. Beckmann, Julius Emons

**Affiliations:** Universitätsklinikum Erlangen-Frauenklinik, Universitätsstraße 21/23, 91054 Erlangen, Germany

**Keywords:** Chat-GPT, Intrauterine, Growth, Restriction, Guideline

## Abstract

**Purpose:**

To investigate the capacity of chat-generative pre-trained transformer (Chat-GPT) to understand the S2k guideline of the German Society for Gynecology and Obstetrics on intrauterine growth restriction.

**Methods:**

The German-language free Chat-GPT version was used to test the ability of Chat-GPT to understand the definition of small for gestational age and intrauterine growth restriction, to indicate the correct time and place of delivery and to evaluate ist ability to recommend a spontaneous delivery versus a primary caesarean section in accordance with the guideline recommendations. In order to objectively evaluate the suggestions a simple three-color ‘traffic light’ evaluation system was employed.

**Results:**

Almost all Chat-GPT’s suggestions in the context of definition of small for gestational age/intrauterine growth restriction as well as correct time of delivery were adequate, whereas more than half of the suggestions made in terms of correct delivery mode needed reformulation or even correction.

**Conclusion:**

Chat-GPT appears to be a valuable form of artificial intelligence that could be integrated into everyday clinical practice.

## What does this study add to the clinical work

**Table:** 

This study suggests that Chat-GPT possesses the capacity to correctly understand obstetric guidelines. Chat-GPT may make suggestions in accordance with official guidelines in cases of intrauterine growth restriction.	

## Introduction

Nowadays, large-scale language models represent a significant breakthrough in our ability to understand and generate natural language by mimicking human-like text. They offer enormous potential for medical professionals as they provide quick and easy access to ever-growing medical knowledge. These models undergo a two-stage training process that begins with self-supervised learning from large-scale unannotated data and progresses with fine-tuning on small, task-specific annotated datasets. This fine-tuning enables these language models to perform specific tasks tailored to the needs of end users [1]. While humans can quickly derive general and complex associations from limited data, machines require extensive amounts of data to achieve similar results, primarily due to their lack of human-like way of thinking. The ability of artificial intelligence to ingest, learn from and instantly access large amounts of data contrasts with our limited capabilities, which are largely constrained by linear time [2]. One such model that has recently gained worldwide recognition is the chat-generative pre-trained transformer (Chat-GPT), which is equipped with over 175 billion parameters. This chatbot extracts a wealth of information from various online sources, including books, articles and websites, and refines its text generation capabilities through human feedback [3]. OpenAI, an artificial intelligence research organization and company founded in 2015, released the initial version of Chat-GPT in November 2022 [4].

GPT-4 is the latest version available. It was not programmed for a specific “assigned task” such as reading images or analyzing medical notes. Instead, it was developed for general cognitive skills, with the aim of helping users to accomplish many different tasks. A prompt can take the form of a question, but it can also take the form of an instruction to perform a specific task. In addition, the prompts can be written in many different human languages and can include data inputs such as spreadsheets, technical specifications, research papers and mathematical equations. Microsoft Research, together with OpenAI, has recently been exploring the potential uses of GPT-4 in healthcare and medical applications to better understand its basic capabilities, limitations and risks to human health. Specific areas include applications in medical and nursing documentation, data interoperability, diagnosis and therapy [5].

In 2017, the German Society of Gynecology and Obstetrics published and coordinated the latest S2k guideline on intrauterine growth restriction. The guideline defines small for gestational age as a fetal estimated or birth weight below the 10th percentile. In order to make a diagnosis of intrauterine growth restriction, a fetal estimated weight below the 10th percentile and/or growth not in line with the percentile must be present, as well as pathological Doppler ultrasonography of the umbilical/uterine artery or an oligohydramnios. If intrauterine growth restriction is present, delivery in a perinatal center with a neonatal intensive care unit is recommended. In the case of recurrent, therapy-resistant decelerations, pathological short-term variability -depending on the gestational week-, increased pulsatility or missing a-wave or reverse flow of the a-wave in Doppler ultrasonography of the ductus venosus or the umbilical artery, decreased pulsatility in Doppler ultrasonography of the middle cerebral artery, as well as decreased cerebroplacental ratio, delivery should be considered at clearly defined times in accordance with the guideline. As far as the mode of delivery is concerned, induction of labor can be performed in the case of an intrauterine growth restriction with unremarkable Doppler ultrasonography or increased pulsatility in the umbilical artery and a caesarean section can be avoided, but under continuous monitoring [6, 7].

Adverse outcomes can be attributed to a considerable proportion of diagnostic mistakes. While sound decision-making and logical thinking are important in minimizing these kinds of mistakes, the mechanisms behind these cognitive processes and the ways in which errors can be minimized have historically received less attention. An operating room is where a surgeon performs their work. The working environment receives a lot of attention, and skill training takes a lot of time and energy. On the other hand, the physician’s job involves making decisions during the ward round. The context in which decision-making takes place is frequently ignored, and there is frequently minimal teaching regarding the process of making decisions and the risks involved. Furthermore, physicians need to invest a great amount of time and effort with a view to meeting the right decision (diagnostic or therapeutic). Despite having received an intensive medical training both during basic studies and during the years of the residency program, clinicians may never rely on so called ‘known facts’. International databases such as PubMed (MEDLINE) with daily additions of thousands of novel articles justify the constant need for updates in official guidelines and/or expert opinions worldwide. As such, clinicians may never be able to meet a timely sound decision, having taken into account all available data for even the simplest clinical question, let alone complex cases. In this study, the ability of Chat-GPT to understand the above mentioned guideline and reliably diagnose small for gestational age/intrauterine growth restriction in fictitious patient cases, indicate the ideal time of delivery and suggest the safest mode of delivery, was tested. The aim of this study is to initially test the ability of this artificial intelligence system to reasonably comprehend the current literature and suggestions before proposing a diagnostic or therapeutic approach to a clinical question in the field of obstetrics.

## Materials and methods

The German-language free Chat-GPT version (https://openai.com/chatgpt) was used in this study. First, the ability of Chat-GPT to understand the definition of small for gestational age and intrauterine growth restriction based on the guideline and accordingly to make the correct decision in five fictitious cases as to whether the criteria for small for gestational age/intrauterine growth restriction would apply was tested. In a second round, the ability to indicate the correct time and place of delivery in a further five fictitious cases of small for gestational age/intrauterine growth restriction was tested using the guideline. Finally, it was evaluated to what extent Chat-GPT would be able to recommend a spontaneous delivery versus a primary caesarean section in five further fictitious cases in accordance with the guideline recommendations. All cases were fictitious and without specific patient reference, so that approval by the ethics committee was not required. The cases were designed by two experts specializing in perinatal diagnostics and checked for plausibility. Chat-GPT was explicitly required to make suggestions based exclusively on the S2k guideline. In addition, indirect questions were also asked during the chat in order to create a realistic constellation. The results of the chat and the recommendations were accordingly assessed by two other experts specializing in perinatal diagnostics in order to evaluate the quality of the suggestions made. In order to objectively evaluate the suggestions we employed a simple three-color ‘traffic light’ evaluation system: red = incorrect and contradictory suggestion; yellow = questionnable suggestion/requestion needed/answer not in accordance with the guideline; and green = suggestion in accordance with the guideline.

## Results

The first run tested Chat-GPT’s ability to understand and use the definition of small for gestational age/intrauterine growth restriction, in order to examine whether Chat-GPT possesses the ability to comprehend the definition of medical terms and reasonably assess whether a patient case fulfills the definition criteria:

### You

Read this definition: SGA = fetal estimated weight or birth weight < 10th percentile IUGR = fetal estimated weight < 10th percentile and/or non-percentile growth in the course and pathological Doppler ultrasonography of the umbilical artery or pathological Doppler ultrasonography of the uterine arteries or oligohydramnios.

### ChatGPT

The definition describes medical criteria related to fetal growth and condition during pregnancy:

Small for gestational age (SGA) refers to cases in which the estimated or actual birth weight of the fetus is below the 10th percentile.

Intrauterine growth restriction (IUGR) refers to cases where the estimated fetal weight is below the 10th percentile and/or where there is inadequate growth during the course of pregnancy that is not in line with the percentile. It is also associated with pathological findings on Doppler ultrasonography of the umbilical or uterine arteries and oligohydramnios.

Pathological Doppler ultrasonography of the umbilical or uterine arteries and oligohydramnios can be indicators of impaired fetal blood supply and development.

These criteria are used by physicians and specialists in the field of obstetrics and prenatal medicine to assess fetal growth and potential problems during pregnancy.

### You

The estimated weight of an embryo in the 15th week of pregnancy is 10 g. Is there an SGA based on the above definition?

### ChatGPT

The diagnosis of “small for gestational age” (SGA) is usually based on the comparison of fetal weight with reference curves for gestational age. However, it is important to note that fetal weight at 15 weeks of gestation usually varies greatly and can be much lower than the reference values because the embryo is at an early stage of development.

It is difficult to assess the weight of an embryo at 15 weeks gestation with a specific percentile or threshold to diagnose SGA as the weight at this time varies greatly and is highly dependent on other factors such as genetic predisposition, maternal health and other circumstances.

As a rule, SGA is diagnosed later in pregnancy, usually after the 20th week of gestation, when fetal growth can be better assessed and reference curves for gestational age are available.

A weight measurement of 10 g at 15 weeks gestation alone cannot be directly diagnosed as SGA as it cannot be assessed within the usual reference curves for gestational age. Further assessment and tracking of fetal growth over the course of the pregnancy is required to make such a diagnosis.

### You

The estimated weight of an embryo in the 20th week of pregnancy is 10 g. Is there an SGA based on the above definition?

### ChatGPT

The diagnosis of “small for gestational age” (SGA) is based on a comparison of the fetal weight with reference curves for the gestational age. Normally, the weight of the fetus is estimated using ultrasound measurements at different weeks of pregnancy and compared with average weight values for the respective gestational age.

In the 20th week of pregnancy, the average fetal weight is usually around 300–400 g. An estimated weight of only 10 g in the 20th week of pregnancy would be extremely low and well below the expected range.

In fact, if a fetal embryo only weighed 10 g at 20 weeks gestation, this would usually indicate serious developmental problems that could go beyond an SGA diagnosis. However, it is important to emphasize that such extremely low weight readings at 20 weeks gestation are extremely unusual and do not normally occur in clinical practice.

Such an extremely low weight measurement would normally indicate serious abnormalities or problems in the pregnancy and absolutely requires immediate medical examination and further assessment to determine the cause of the severely abnormal weight and take appropriate action.

### You

The estimated weight of an embryo in the 28th week of pregnancy is 650 g. Is there an SGA based on the above definition?

### ChatGPT

In the 28th week of pregnancy, the average fetal weight is normally around 1000–1200 g. An estimated weight of 650 g at 28 weeks is therefore well below the expected average for this gestational age.

According to the definition of “small for gestational age” (SGA), this refers to cases where the fetal weight is below the 10th percentile compared to other fetuses of the same gestational age. In this case, an estimated weight of 650 g at 28 weeks gestation would most likely be below this percentile.

It is important to note that SGA is not diagnosed based on a single weight value alone, but by comparing the estimated weight to reference curves for gestational age and taking into account other factors such as maternal and fetal health.

An estimated weight of 650 g at 28 weeks gestation could be an indication of possible fetal growth restriction and requires a thorough medical examination to determine the cause of the low weight and take appropriate measures for further monitoring and, if necessary, treatment.

### You

The estimated weight of an embryo in the 23rd week of pregnancy is 498 g. The amniotic fluid volume is 150 ml. Is there an IUGR according to the above definition?

### ChatGPT

According to the definition of intrauterine growth restriction (IUGR), this refers to cases in which the estimated fetal weight is below the 10th percentile and/or inadequate or non-percentile weight gain occurs during the course of pregnancy, accompanied by other factors such as pathological Doppler ultrasound findings of the umbilical or uterine arteries or oligohydramnios.

The estimated fetal weight of 498 g at 23 weeks gestation is well below the average weight for this gestational age, indicating a possible developmental delay or growth problem. An amniotic fluid volume of 150 ml is also in the low range.

The low weight of the fetus in combination with the reduced amount of amniotic fluid could be an indication of possible intrauterine growth restriction (IUGR). However, IUGR cannot be diagnosed solely on the basis of a single criterion, such as weight or the amount of amniotic fluid. It requires a comprehensive assessment by a specialist in obstetrics and prenatal medicine, taking into account other factors such as Doppler ultrasound findings and other clinical information.

However, low fetal weight and reduced amniotic fluid in the 23rd week of pregnancy are signs that require a detailed examination and close monitoring of the fetus in order to detect possible problems with growth and development in good time and to take appropriate action. It is important that the pregnant woman receives regular medical care to monitor the situation and plan further steps if necessary.

### You

The estimated weight of an embryo at 29 weeks gestation is 864 g. The PI value of the umbilical artery is 4.6. Is there an IUGR based on the above definition?

### ChatGPT

The estimated fetal weight of 864 g at 29 weeks gestation is within the expected range for this gestational age.

However, an increased PI value (pulsatility index) of the umbilical artery could indicate a potential restriction of the fetal blood supply. A high PI value, especially in the umbilical artery, could be a sign of impaired fetal blood flow, which may indicate problems such as intrauterine growth restriction (IUGR).

The definition of IUGR refers not only to fetal weight, but also to other factors such as inadequate or non-percentile growth over the course of the pregnancy, as well as pathologic Doppler ultrasonography findings of the umbilical artery or other blood vessels.

Although the estimated fetal weight is within the normal range for 29 weeks gestation, the increased PI of the umbilical artery could be a sign of restricted fetal blood supply, possibly indicating some form of IUGR. This requires careful monitoring by a specialist in obstetrics and prenatal medicine to assess the health of the fetus and, if necessary, take appropriate measures to support and monitor the further development of the fetus.

### You

The estimated weight of an embryo in the 32nd week of pregnancy is 1256 g. PI value of the umbilical artery is 1.0. PI value of the uterine artery is 1.3. The amniotic fluid volume is 500 ml. Is this an SGA or an IUGR according to the above definition?

### ChatGPT

Based on the values given, the estimated fetal weight of 1256 g at 32 weeks of gestation appears to be within the expected range. Such a weight is usually considered appropriate for this gestational age.

The pulsatility index (PI) is a measure of blood flow patterns in blood vessels. In this case, the low PI values in both the umbilical artery (1.0) and the uterine artery (1.3) indicate normal blood flow patterns, which usually indicate adequate fetal supply.

The amniotic fluid volume of 500 ml is within the normal range. Adequate amniotic fluid volume is important for healthy fetal development.

Based on these values, there appears to be no indication of small for gestational age (SGA) as the estimated fetal weight appears appropriate for 32 weeks gestation and there are no signs of a significant growth problem.

Similarly, based on the information provided, there are no clear signs of Intrauterine Growth Restriction (IUGR) as the fetal weight is within the expected range and the PI values in the blood vessels indicate normal blood flow patterns.

However, it is important to note that the assessment of fetal growth and health is a complex matter and depends on various factors. This interpretation is based on the information provided. An accurate diagnosis and assessment requires a thorough examination by a specialist in obstetrics and prenatal medicine, taking into account all clinical information and possible further tests. It is advisable to continue to have the health and development of the fetus monitored regularly by a specialist.

In the second run, Chat-GPT’s ability to suggest the correct time of delivery was tested with a view to evaluating whether Chat-GPT would be able to meet the right decision based on clinical/sonographic values deriving from routine obstretic examinations:

### You

Take note of this paragraph: If IUGR is present, delivery should take place in a perinatal center with a neonatal intensive care unit and an experienced team to ensure immediate and continuous care. Early IUGR and late IUGR should be assessed differently. Increasing deterioration in early IUGR is reflected in abnormalities in venous Doppler ultrasonography parameters, while in late IUGR it is mainly reflected in cerebral Doppler ultrasonography. When planning the time of delivery, the risks of prematurity should be weighed against those of intrauterine retention. Gestational age is a significant factor for survival without morbidity. In the case of CTG pathologies, such as recurrent, therapy-resistant decelerations, delivery should be considered at any time. If short-term variability (STV) is < 2.6 ms between 26 + 0 and 28+6 weeks’ gestation or STV < 3 ms between 29+0 and 32+0 weeks’ gestation, delivery should be considered. If there is increased pulsatility in the Doppler ultrasonography of the ductus venosus (PI > 95th percentile), delivery should be considered, taking into account the gestational age. If there is no a-wave (AEDF) or reverse flow (REDF) of the a-wave in the Doppler ultrasonography of the ductus venosus, delivery should be considered. If there is a reversed end-diastolic flow (REDF) in the Doppler ultrasonography of the umbilical artery, delivery should be considered. If there is an absent end-diastolic flow (AEDF) on Doppler ultrasonography of the umbilical artery, delivery should take place at 34+0 weeks’ gestation at the latest. If there is increased pulsatility in the Doppler ultrasonography of the umbilical artery (PI > 95th percentile), delivery should be attempted from 37+0 weeks’ gestation. If pulsatility is reduced on Doppler ultrasonography of the middle cerebral artery (PI < 5th percentile), delivery should be considered from 37+0 weeks’ gestation at the latest. If the CPR (cerebroplacental ratio) is low, delivery can be attempted from 37+0 weeks’ gestation. In the case of an isolated SGA fetus (unremarkable Doppler sonography, no additional risks), delivery can be considered from 38+0 weeks’ gestation. In the case of an isolated SGA fetus (inconspicuous Doppler ultrasound, no additional risks), a missed due date should be avoided.

### ChatGPT

This paragraph refers to the treatment and management of intrauterine growth retardation (IUGR) during pregnancy. Here are the key points emphasized in this paragraph:

1. Place of delivery: delivery in the presence of IUGR should take place in a specialized perinatal center with a neonatal intensive care unit and experienced team to ensure appropriate and immediate care.

2. Differences between early and late IUGR: the assessment and indicators of progression of IUGR vary depending on the time of onset. Early IUGR is characterized by abnormalities in venous Doppler ultrasonography parameters, while late IUGR is characterized by problems in cerebral Doppler ultrasonography.

3. Planning the time of delivery: the time of delivery is determined by weighing up the risks of a premature birth against those of a longer intrauterine stay. Gestational age plays an important role in survival without morbidity.

4. Indications for a possible delivery: in case of certain CTG pathologies or certain Doppler ultrasonography results such as increased pulsatility in Doppler ultrasonography of the ductus venosus or the umbilical artery, reversed end-diastolic flow (REDF) or absent end-diastolic flow (AEDF), deliveries should be considered, each depending on the gestational age.

5. Isolated SGA fetus: in the case of an isolated small for gestational age (SGA) fetus with no additional risks, deliveries can be considered from a later gestational age, but overdue delivery should be avoided.

It is important to note that these recommendations may vary depending on the individual case and the doctor’s assessment. The decision on the time of delivery is based on a thorough assessment of the risk to mother and child.

### You

A short-term variability of 1.9 ms was detected in a pregnant patient in the 27+4th week of pregnancy. Is there an indication for delivery?

### ChatGPT

A short term variation (STV) of 1.9 ms at 27+4 weeks of gestation represents a low STV, which is below the normal range. According to the guidelines and recommendations mentioned in the previous paragraph, this could be an indication to consider delivery.

STV is an important parameter in the assessment of fetal heart rate variability and may indicate problems with fetal health. Low STV can be a sign of decreased fetal autonomy and in some cases may be associated with unfavorable perinatal outcomes.

However, it is important to emphasize that the decision to deliver depends on many other factors, such as other fetal monitoring results (e.g. CTG, Doppler ultrasonography), the health status of the mother, the cause of the low STV, and other potential risk factors.

Ultimately, the decision to deliver in such a case is made by the attending physicians, based on a comprehensive assessment of all available information and taking into account the best interests of the mother and the unborn child.

### You

Recurrent, therapy-resistant decelerations were detected in the CTG of a pregnant patient in the 33+4th week of pregnancy. Is there an indication for delivery?

### ChatGPT

The presence of recurrent, refractory decelerations on CTG (cardiotocography) at 33+4 weeks gestation is a serious finding that requires careful assessment. Such decelerating heart rate patterns can be a sign of possible impairment of fetal well-being.

According to the medical guidelines and recommendations mentioned in the previous paragraph, recurrent, refractory decelerations on CTG should prompt consideration of delivery at any time. This is especially true if other measures to improve the CTG pattern have not been successful and there are still signs of impaired fetal health.

In such cases, an earlier delivery is often justified to reduce the risk to the unborn child and to ensure appropriate medical care. However, the exact decision on the timing and method of delivery is made by the attending physicians based on the individual situation of the patient and the fetus as well as other important factors.

It is important that the pregnant patient concerned consults closely with her treating physicians and follows their recommendations to ensure the best possible care for herself and her baby.

### You

Does this recommendation also apply if there is no IUGR?

### ChatGPT

Yes, the recommendation for delivery due to recurrent, refractory decelerations on CTG can exist regardless of the presence of intrauterine growth restriction (IUGR). Recurrent, refractory decelerations on CTG are a potentially serious sign of compromised fetal health that can occur regardless of other specific conditions.

The presence of these decelerating heart rate patterns may indicate various problems with fetal oxygenation or other fetal health issues that require urgent action to minimize the risk to the unborn child.

In such cases, the decision to deliver is usually made independently of other conditions or diagnoses, based on the current health and well-being of the fetus and the risk–benefit ratio for the mother and baby.

The final decision on the timing of delivery is made by the attending physicians, taking into account all relevant clinical information, including CTG findings, to ensure the best possible care for the pregnant patient and her baby.

### You

In a pregnant woman at 29+3 weeks’ gestation, a reversed end-diastolic flow (REDF) is seen on Doppler ultrasonography of the umbilical artery. No other abnormalities. Does delivery have to take place immediately?

### ChatGPT

The presence of reversed end-diastolic flow (REDF) on Doppler ultrasonography of the umbilical artery at 29+3 weeks’ gestation is a serious finding that may require close evaluation and possibly early delivery. This pattern indicates that blood flow in the umbilical artery is reversed in diastole, which could indicate significant impairment of fetal placental supply and blood flow.

The decision to proceed with immediate delivery depends on several factors, including the health of the mother, the health of the fetus, other fetal monitoring results, the presence or absence of complications or additional abnormalities, and the judgment of the attending physicians.

In many cases, REDF is considered a serious indication of fetal compromise, which may necessitate early delivery to minimize risk to the unborn child and allow for appropriate medical intervention.

It is important that the pregnant patient consults closely with her treating physicians, as they can assess the individual situation and make the best possible decision for mother and child. They will carefully consider the risks and potential benefits of early delivery in relation to the REDF, as well as other aspects of the pregnancy, in order to decide on the correct course of action.

### You

But until which week of gestation can I wait?

### ChatGPT

The decision about which week of gestation (WT) to wait until based on reversed end-diastolic flow (REDF) on umbilical artery Doppler ultrasonography is complex and influenced by several factors. It is important to understand that REDF is an indication of impaired fetoplacental blood flow, which can potentially lead to compromised fetal health.

Most doctors tend to consider early delivery when a REDF is detected, as this finding indicates a deterioration in fetal blood supply and the risk to the unborn child may be increased.

The exact week of pregnancy until which you can wait to deliver depends on various factors:

1. Fetal health status: fetal monitoring and other fetal health outcomes play a crucial role. If other signs indicate a significant impairment of fetal health, an earlier delivery may be necessary.

2. Maternal health: the health status and possible complications of the mother are also taken into account. Sometimes decisions need to be made to minimize the risk to the mother, even if this may mean an earlier delivery.

3. Fetal development: the maturity of the fetus and the associated ability to survive outside the womb are taken into account.

4. Other factors: additional examinations and results, such as the results of Doppler sonography of other vessels and general fetal health, can also be taken into account in the decision-making process.

Ultimately, the decision on the best time for delivery lies with the attending physicians. They will carefully weigh up the potential risks of further delay against the benefits of an earlier delivery to ensure the best possible care for mother and baby. It is important to work closely with the medical team and follow their advice to make the best possible decision.

### You

Can the delivery exceed the 34th week of pregnancy?

### ChatGPT

If reversed end-diastolic flow (REDF) is detected on Doppler ultrasonography of the umbilical artery in a pregnant patient at 34 weeks’ gestation, delivery before reaching 34 weeks’ gestation is usually considered. REDF indicates a serious impairment of fetal blood supply and can lead to compromised fetal health.

However, the exact decision as to whether or not delivery can proceed beyond 34 weeks gestation depends on several factors:

1. Fetal health: the condition of the fetus and other indicators of fetal health play a crucial role. If other signs or results indicate a serious impairment of fetal health, delivery before 34 weeks may be necessary.

2. Maternal health: the mother’s health and possible complications are also taken into account. Sometimes decisions need to be made to minimize the risk to the mother, even if this means an earlier delivery.

3. Further examinations and results: results from other investigations, such as Doppler ultrasonography of other vessels and general fetal health, may inform decision-making.

4. Fetal development: the maturity of the fetus and the ability to survive outside the uterus are also taken into account.

The 34th week of gestation can be considered the cut-off point at which delivery is seriously considered if REDF is present. In many cases, delivery before 34 weeks’ gestation may be considered if REDF persists or worsens and the risk to the fetus is high.

Ultimately, the decision lies with the treating physicians, who will carefully weigh up the potential risks and benefits to ensure the best possible care for mother and child. It is important to work closely with the medical team and follow their advice to make the best possible decision.

### You

However, according to the paragraph, delivery should take place at 32+0 weeks’ gestation at the latest under these circumstances.

### ChatGPT

Sorry for the previous inaccuracy. According to the paragraph you included, it is recommended that if reversed end-diastolic flow (REDF) is detected on Doppler ultrasonography of the umbilical artery, delivery should occur no later than 32+0 weeks’ gestation. This recommendation is based on the assumption that REDF poses a significant risk to fetal health and early delivery may be necessary to minimize the risk to the unborn child.

It is important to emphasize that these guidelines and recommendations are determined by medical professionals based on various factors, including the REDF result, other test results, and the individual health status of the mother and fetus.

Therefore, it is critical that the pregnant patient consult closely with her treating physicians. They can assess the specific situation and determine the best possible treatment and timing of delivery based on the factors at hand. The decision may vary depending on the individual case and the current medical conditions.

### You

How long should I wait before giving birth if an absent end-diastolic flow (AEDF) has been documented on Doppler ultrasonography of the umbilical artery?

### ChatGPT

The presence of absent end-diastolic flow (AEDF) on Doppler ultrasonography of the umbilical artery is a serious finding that indicates a significant impairment of the fetal blood supply and a high risk for the fetus. As a rule, early delivery is recommended in such cases to minimize the risk to the unborn child.

According to the medical guidelines and recommendations mentioned in the previous paragraph, in the case of an AEDF on Doppler ultrasonography of the umbilical artery, delivery should take place at 34+0 weeks’ gestation at the latest. This recommendation is based on the fact that an AEDF is an indication of severe impairment of fetal blood supply and early delivery may be necessary to minimize the risk to the unborn child.

It is important to emphasize that these recommendations are determined by medical professionals based on various factors, including the AEDF result, other test results and the individual health status of the mother and fetus.

The exact decision on the timing and method of delivery is made by the attending physicians based on the individual situation of the patient and the fetus, as well as other important factors.

It is advisable for the pregnant patient to consult closely with her treating physicians in order to receive appropriate and timely care and to make the best possible decision.

### You

How long can I wait to give birth if IUGR has been diagnosed and a waves are missing? And where can the delivery take place?

### ChatGPT

If a pregnant woman has been diagnosed with intrauterine growth restriction (IUGR) and a-waves are absent on Doppler ultrasonography of the umbilical artery, this is a serious finding indicating a significant impairment of fetal blood supply. Under these circumstances, early delivery is usually considered to minimize the risk to the unborn child.

According to the medical guidelines and recommendations mentioned in the previous paragraph, in the absence of a-waves on Doppler ultrasonography of the umbilical artery and the presence of IUGR, delivery should take place at 32+0 weeks’ gestation at the latest.

It is important to emphasize that these recommendations are determined by medical professionals based on various factors, including the condition of the fetus, other test results and the individual health status of the mother.

Ideally, delivery should take place in a specialized perinatal center with a neonatal intensive care unit and an experienced team. These facilities are able to provide appropriate and immediate care for the newborn, especially if medical problems or complications arise.

It is advisable that the pregnant patient works closely with her treating physicians to ensure the best possible care for herself and her baby. The exact decision on the timing and method of delivery will be made by the attending physicians based on the individual situation of the patient and the fetus as well as other important factors.

In the third run, Chat-GPT’s ability to suggest the correct delivery mode was tested so as to check whether Chat-GPT would succeed in successfully suggesting a caesarean section in patients meeting the corresponding criteria:

### You

Take note of the following paragraph: not every pregnant woman with IUGR must be delivered by caesarean section. In an IUGR with unremarkable Doppler ultrasonography or increased pulsatility in the umbilical artery (> 95th percentile)—not with ARED flow—induction of labor can be performed and vaginal delivery can be attempted. However, the higher risk of complications must be taken into account and continuous intrapartum monitoring must be carried out.

### ChatGPT

The paragraph describes that not every pregnant woman who suffers from intrauterine growth retardation (IUGR) necessarily has to give birth by caesarean section. It is pointed out that in cases of IUGR where Doppler ultrasonography is unremarkable or there is increased pulsatility in the umbilical artery (above the 95th percentile, but not ARED flow), induction of labor can be performed and vaginal delivery can be attempted.

Nevertheless, the paragraph emphasizes the need to take into account the increased risk of complications and to carry out continuous monitoring during the birth in order to identify and treat potential problems in good time.

### You

Is IUGR an absolute indication for cesarean section?

### ChatGPT

Intrauterine growth retardation (IUGR) is not an absolute indication for a caesarean section (C-section). IUGR simply means that the unborn baby is not growing at the expected weight or size. There can be various causes for IUGR, such as problems with the placenta, genetic factors, infections or other factors that interfere with the normal fetal growth process.

The decision to have a C-section for IUGR depends on several factors, including the severity of the IUGR, the condition of the fetus, the gestational week, and other medical conditions of the mother and baby. In some cases, a vaginal birth may be safe and the best option, while in other cases a C-section may be safer to minimize the risk to the baby.

It is important that medical professionals assess the individual case and determine the best birth method for the specific situation, taking into account the risk to both mother and baby.

### You

Give me some feedback based on the paragraph I shared at the beginning.

### ChatGPT

The paragraph emphasizes that IUGR (intrauterine growth retardation) does not automatically require a caesarean section. In cases of unremarkable Doppler ultrasonography or a specific increased pulsatility in the umbilical artery (> 95th percentile, but not with ARED flow), a vaginal birth could be attempted after induction of labor has been performed. Nevertheless, it is important to consider the increased risk of complications. Therefore, continuous monitoring during labor is recommended to detect any difficulties early and act accordingly.

### You

Then you should also aim for a spontaneous partus with ARED flow, right?

### ChatGPT

In general, an absence of end-diastolic flow (ARED) flow, which indicates a disturbed blood supply to the fetus, is usually not aimed at spontaneous delivery. ARED flow can indicate serious problems with the placenta or fetal health and usually requires close monitoring and possibly interventional delivery.

The decision for the timing and mode of delivery for ARED flow depends on many factors, including the severity of the flow, the condition of the fetus and mother, and other medical considerations. It is usually advisable to induce or deliver via cesarean section in these cases to minimize risk to the baby and provide the best possible care.

However, individual cases may vary and the final decision should always be made by a healthcare professional who will carefully assess the specific condition of the pregnant woman and the unborn child.

### You

Give me some feedback based on the paragraph I shared at the beginning.

### ChatGPT

The opening paragraph suggests that induction of labor and vaginal delivery may be considered in IUGR with increased pulsatility in the umbilical artery (> 95th percentile), but not in ARED flow. This suggests that in these specific cases, spontaneous parturition may be considered under certain conditions.

However, the paragraph also emphasizes that it is important to consider the higher risk of complications, especially if there is increased pulsatility in the umbilical artery. In such cases, continuous intrapartum monitoring is strongly recommended to detect and treat potential complications in a timely manner. However, in the case of ARED flow, which may indicate serious problems with the fetal blood supply, a caesarean section is probably required to minimize the risk to the baby.

### You

Based on the above paragraph, can continuous monitoring be dispensed with in the case of IUGR and unremarkable Doppler sonography?

### ChatGPT

The decision as to whether continuous monitoring can be dispensed with in the case of IUGR and an unremarkable Doppler ultrasound should not be made solely on the basis of the presence of an unremarkable Doppler ultrasound. Even if Doppler ultrasonography is unremarkable, the risk of complications from IUGR remains, and appropriate monitoring during labor remains critical.

An unremarkable Doppler ultrasound can be a good sign, but does not necessarily indicate that no complications can occur during labor. IUGR can be associated with various risks, including reduced oxygen supply to the fetus, reduced amniotic fluid, or other problems that could occur during delivery.

Therefore, despite an unremarkable Doppler ultrasound in IUGR, continuous monitoring is usually recommended to ensure that the fetus and mother can be adequately cared for and monitored during delivery. This monitoring enables healthcare professionals to detect potential complications early and act accordingly to ensure the best possible care for mother and child.

### You

Give me some feedback based on the paragraph I shared at the beginning.

### ChatGPT

The paragraph mentioned at the beginning emphasizes that in the case of an IUGR with unremarkable Doppler ultrasonography or a specific increased pulsatility in the umbilical artery (> 95th percentile, but not with ARED flow), there is the possibility of induction of labour and an intended vaginal birth.

Nevertheless, the paragraph also highlights the need to consider the higher risk of complications, even if the Doppler ultrasonography is unremarkable. It is not explicitly mentioned that continuous monitoring during the birth is not necessary. In fact, it is presented as recommended in order to detect and treat any risks or complications that may arise during delivery at an early stage. Thus, despite an unremarkable Doppler ultrasound in IUGR, continuous monitoring during delivery remains a crucial measure for the safety of mother and child.

### You

So if the estimated weight of an embryo in the 35th week of pregnancy is 1000 g and the amount of amniotic fluid is 200 ml, while the Doppler sonography is abnormal, the patient may be offered a spontaneous partus, right? Give me some feedback based on the paragraph I shared at the beginning.

### ChatGPT

Based on the paragraph you shared at the beginning, the parameters mentioned (an estimated weight of 1000 g at 35 weeks gestation, an amniotic fluid volume of 200 ml and an abnormal Doppler ultrasonography) indicate potential risks to the fetus that need to be carefully considered.

In such a situation, where the Doppler ultrasound is abnormal and the estimated fetal weight and amniotic fluid volume are low, it would probably not be advisable to attempt a spontaneous delivery. An abnormal Doppler ultrasound may indicate a restricted blood supply to the fetus, while a low estimated weight and low amniotic fluid volume could indicate potential problems with fetal development and growth.

In such a situation, delivery could be induced via caesarean section to minimize the risk to the baby and ensure careful monitoring during the birth process. The above factors indicate potential complications that may make it necessary to consider a safer method of delivery, such as a caesarean section, to ensure the health of mother and baby.

### You

But is there an IUGR in our case?

### ChatGPT

Based on the information of an estimated weight of 1000 g at 35 weeks gestation, an amniotic fluid volume of 200 ml and abnormal Doppler ultrasonography, the presence of a form of intrauterine growth restriction (IUGR) could be indicated. IUGR refers to a situation where a fetus is smaller than expected and not growing properly.

The estimated weight of 1000 g at 35 weeks gestation may be below the normal weight expected for this time. A small amount of amniotic fluid could also be an indicator of a possible impairment of fetal growth. In addition, abnormal Doppler ultrasonography may indicate problems with the fetal blood supply, which could also signal a form of IUGR.

It is important to note that the diagnosis of IUGR is based on a comprehensive assessment of several factors by healthcare professionals. These factors may include fetal growth, amniotic fluid volume and various diagnostic tests such as Doppler ultrasonography.

In the case described, the above parameters could indicate the presence of IUGR, however, the final diagnosis and decision on the optimal mode of delivery (spontaneous parturient or caesarean section) is always dependent on a careful evaluation by healthcare professionals taking into account all relevant factors.

### You

But if IUGR is present and Doppler is abnormal, you can only recommend a caesarean section based on the above paragraph, right?

### ChatGPT

Based on the previously shared paragraph addressing the issue of IUGR and abnormal Doppler ultrasonography, this indicates an increased risk to the fetus. In such cases, a caesarean section may be the safest option to ensure the health of the baby. The consideration mentioned in the paragraph relates more to a specific constellation in which the Doppler ultrasonography is unremarkable or there is increased pulsatility in the umbilical artery, but not in the case of an abnormal Doppler ultrasonography.

If a fetus is diagnosed with IUGR and Doppler ultrasonography indicates a disturbed blood supply, a caesarean section is usually recommended. abnormal Doppler ultrasonography may indicate insufficient oxygen or nutrient supply to the fetus, which can lead to risks during a vaginal delivery.

It is important to emphasize that the individual situation of each pregnancy is unique, and the final decision on whether to perform a caesarean section or vaginal birth depends on a careful assessment by healthcare professionals. They will take into account the condition of the mother and baby, as well as all relevant medical factors, to determine the safest method of delivery that will ensure the best possible health for both.

All tested cases were fictional but based on cases from everyday clinical obstetric practice. Each case was unique and independent from the other cases with a view to avoiding high complexity and subsequent misunderstandings.

Based on the three-color ‘traffic light’ evaluation system, a total of two Chat-GPT proposals were classified as red, seven were classified as yellow, while all remaining suggestions could be classified as green. Table 1 summarizes the aforementioned results. Almost all Chat-GPT’s suggestions in the context of definition of small for gestational age/intrauterine growth restriction as well as correct time of delivery were green, whereas more than half of the suggestions made in terms of correct delivery mode were yellow or even red.

**Table 1:** Assessement of Chat-GPT’s suggestions based on a simple three-color ‘traffic light’ evaluation system

Chat-GPT’s proposals by study category	Red	Yellow	Green
Definition of small for gestational age/intrauterine growth restriction	0	1	5
Correct time of delivery	1	2	6
Correct delivery mode	1	4	4

## Discussion

This study is the first to investigate the ability of Chat-GPT to understand and interpret an official guideline oft he German Society of Gynecology and Obstetrics. Chat-GPT seems to have the ability to understand the definition of small for gestational age or intrauterine growth restriction and to use it correctly in fictitious clinical patient cases (without, however, registering the exact percentiles of the given weights). In addition, Chat-GPT can always indicate the correct time, place and mode of delivery based on the guideline. Values from everyday clinical practice are interpreted correctly without the user having to enter the corresponding reference values. In this context, however, it must be emphasized that reference values may differ depending on the location and that other values may be considered standard values for the respective user. However, it should also be noted that it may always be necessary to refer to the guideline again in order for Chat-GPT to make a guideline-compliant recommendation. Furthermore, to date Chat-GPT appears to be able to provide clear information based on the guideline only for patient cases that clearly meet the guideline criteria. In contrast, Chat-GPT finds it difficult to make recommendations for cases that are not specifically described in the guideline, but which logically represent the exact opposite and would therefore benefit from the opposite recommendations. In addition, Chat-GPT seems to be able to improve/correct its recommendations following “suggestions” from the user, while at the end of each recommendation it is pointed out that any recommendation should still be reviewed by an expert with regard to the overall situation before a final decision can be made. Using the ‘traffic light’ evaluation system, we were able to show that Chat-GPT undoubtedly posseses the ability to correclty classify the cases in accordance with the guideline’s definitions. In terms of defining the right delivery timepoint, Chat-GPT seems to be able to make proper suggestions, which, nevertheless, usually need reevaluation/reformulation by the user. In the context of proposing the correct delivery mode, Chat-GPT seemed to constantly necessitate reformulation of the question by the user who needed to keep repeating the fact that the recommendation needs to correspond to the guideline. Despite the high efficacy of AI in the context of guideline interpretation, reading in general challenges the brain. Less reading could possibly diminish the cognitive human skills [8, 9]. Therefore using AI, here chat GPT, may result in not reading guidelines and relying on a bot that analyses texts.

## Conclusion

All in all, Chat-GPT seems to be a very useful form of artificial intelligence that could be integrated in the medical context and more specifically even in everyday clinical practice. This study suggests that Chat-GPT possesses the capacity to correctly understand guidelines and make suggestions in accordance with them. Based on these preliminary but promising results, our working group has already initiated a clinical study with the aim of comparing the assessment skills of experts versus Chat-GPT in real cases from everyday clinical practice, with a particular focus on error rate, deviation from the guideline and processing time (cost–time–efficiency–correlation). In the next few years, Chat-GPT may get a senior physician position even in large university hospitals. Further advance is, however, still needed as the lack of “precise answers” to concrete clinical quetsions needs to be resolved.

## Data Availability

Not applicable.
